# Evaluation of the public health laboratory network for tegumentary leishmaniasis in an endemic area of Brazil

**DOI:** 10.1590/S1678-9946202466070

**Published:** 2024-12-06

**Authors:** Fernanda Alvarenga Cardoso Medeiros, Job Alves Souza, Maria Inês Fernandes Pimentel, Ilka Afonso Reis, Daniel Menezes-Souza, Aline Fagundes da Silva, Andreza Pain Marcelino

**Affiliations:** 1 Universidade Federal de Minas Gerais, Faculdade de Medicina, Pós-Graduação em Ciências da Saúde: Infectologia e Medicina Tropical, Belo Horizonte, Minas Gerais, Brazil; 2 Fundação Ezequiel Dias, Instituto Octávio Magalhães, Serviço de Doenças Parasitárias da Divisão de Epidemiologia e Controle de Doenças, Belo Horizonte, Minas Gerais, Brazil; 3 Fundação Oswaldo Cruz, Instituto Nacional de Infectologia Evandro Chagas, Laboratório de Pesquisa Clínica e Vigilância em Leishmaniose, Rio de Janeiro, Rio de Janeiro, Brazil; 4 Universidade Federal de Minas Gerais, Instituto de Ciências Exatas, Belo Horizonte, Minas Gerais, Brazil; 5 Universidade Federal de Minas Gerais, Colégio Técnico, Departamento de Patologia Clínica, Belo Horizonte, Minas Gerais, Brazil

**Keywords:** Tegumentary leishmaniasis, Laboratory diagnosis, Public health laboratories

## Abstract

Diagnostic networks ensure efficiency in disease diagnosis. A descriptive study evaluated the network of public health laboratories (NPHL) in Minas Gerais State, Brazil, using diagnostic results for tegumentary leishmaniasis (TL) from the laboratory management system in 2017–2020. Out of 1,369 individuals analyzed, 704 (51.4%) cases of TL were confirmed, with 610 (86.7%) by direct parasitological examination (DPE) and 94 (13.4%) by polymerase chain reaction (PCR). Notably, 25.3% of cases with DPE-negative results were PCR-positive. Consecutive diagnostic tests enhanced diagnosis of TL. NPHL-MG is a promising model for expanding similar networks and improving disease control.

## INTRODUCTION

Tegumentary leishmaniasis (TL) is endemic in 90 countries, with up to 1,000,000 estimated annual cases worldwide^1^. In Brazil, 12,878 cases of TL were reported in 2022. The Brazilian Minas Gerais State (MG) reported a total of 932 cases of the disease, with 830 cases (89.09%) presenting cutaneous clinical manifestations and 102 cases (10.91%) exhibiting mucosal manifestations, making it the fourth state with the highest number of reported cases in the country^2^. The predominant causative species are *Leishmania (V.) braziliensis* (90%) and *Leishmania (L.) amazonensis* (10%)^2,3^.

The diagnosis of TL is challenging as it clinically resembles several other diseases^4^. Laboratory diagnosis is limited because reference methods are invasive, require qualified professionals, and show low sensitivity^5^. Additionally, the leishmanin skin test (LST), formerly widely used in Brazil, was discontinued in 2016^6^.

Due to the lack of the LST, Ezequiel Dias Foundation (FUNED), Oswaldo Cruz Foundation, and State Health Department of Minas Gerais (SES-MG) issued a technical note^7^ that outlined the structure of the network of public health laboratories (NPHL) for direct parasitological diagnosis of TL in MG. The note established that municipal and regional public reference laboratories are responsible for direct parasitological examination (DPE), while the state central laboratory (FUNED) is responsible for polymerase chain reaction (PCR).

To analyze the performance of the diagnosis of TL in the NPHL in MG after its structuring, a descriptive study was conducted on the diagnostic results for TL from the NPHL in MG recorded in the Gerenciador de Ambiente Laboratorial (GAL – Laboratory Environment Management System) from 2017 to 2020.

## MATERIALS AND METHODS

MG spans 586,513,993 km^2^ and shows a population of 21,411,923 across 853 cities^8^. These cities are organized into eight health regions registered in GAL/MG: 1- Belo Horizonte Macro-region, 2- North, Northeast, Northwest, and Jequitinhonha, 3- Southeast and Centro-South, 4- East, Southeast, and Vale do Aco, 5- West, 6- South, 7- North Triangle and South Triangle, and 8- Belo Horizonte municipality and the metropolitan region.

The technical note on TL diagnosis recommends that for patients suspected of having TL, a direct parasitological examination (DPE) should be initially performed. If this test is negative, a PCR test should be conducted. For cases presenting with the mucosal form of the disease, PCR can be used as the primary test. DPE was conducted following Barcia's methodology^9^ in municipal and regional laboratories within the network. The PCR performed was conventional, qualitative PCR, with DNA revealed via agarose gel electrophoresis at the FUNED laboratory^3^. Parasite DNA and total DNA from biopsy fragments samples were extracted using the QIAamp DNA mini kit (QIAGEN GmbH, Hilden, Germany) following the manufacturer's instructions. Negative DNA extraction controls were performed for each experiment by adding all reagents except the sample. The resulting DNA was resuspended in 100 µL of Milli-Q water. The following primers that amplify the kDNA region of the *Leishmania* genus were used: forward (CCTATTTTACACACACCCCCAGT) and reverse (GGGTAGGGGCGTTCTGCGAAA). The human β-actin gene, using the forward (ACCTCATGAAGATCCTCACC) and reverse (CCATCTCTTGCTCGAAGTCC) primers was used as an endogenous control^10^. Each PCR assay included a negative control (PCR mixture without DNA, a control for the DNA extraction process, and DNA from known negative samples) and a positive control (genomic DNA from *L*. (*V*.) *braziliensis* MHOM/BR/75/M2904 as reference strain).

The key variables analyzed included the results of DPE and PCR, the type of biological samples used for diagnosis, patient profiles (sex and age), and the locations (Health Regions) where the tests were performed.

Results from patients with compatible clinical presentations (single or multiple cutaneous ulcers, multiple nodular and disseminated lesions, and/or mucosal disease) who underwent at least one DPE or PCR test recorded in GAL/MG were eligible for the study. Results from patients confirmed with TL via other methods (serological tests), patients with incorrect biological sample records, or those with limited information were excluded from the study.

Data were summarized as absolute values and percentages, and presented in tables or frequency charts. The study was conducted according to the ethical standards of the Brazilian government (Resolution 466/2012 of the Brazilian National Health Council) and approved by the Research Ethics Committee of Fundacao Ezequiel Dias (protocol Nº CAAE 44042721.5.0000.9507).

## RESULTS

**Figure 1:**
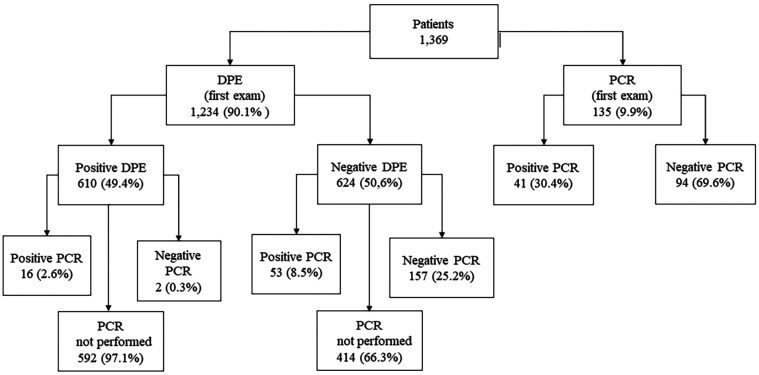
Cutaneous leishmaniasis diagnosis performed in Minas Gerais State public laboratory network. Data on the results of the diagnostic routine from 2017 to 2020 from the Laboratory Environment Management System of Ezequiel Dias Foundation for the diagnosis of cutaneous leishmaniasis; DPE = direct parasitological exam; PCR = polymerase chain reaction.

From 2017 to 2020, NPHL-TL in Minas Gerais State (MG) registered a total of 1,685 diagnostic tests, comprising 1,311 (77.8%) DPE and 374 (22.2%) PCR assays, as recorded in GAL/MG. A cohort of 1,369 individuals suspected of having TL was assessed, leading to the confirmation of 704 cases (51.4%), with an annual average of 172.4 cases. Among the confirmed cases, 610 (86.7%) were identified via DPE, while 94 (13.4%) were confirmed via PCR. Notably, 53 patients (25.3%) with negative DPE results had their samples subjected to PCR testing, which yielded positive results. Figure 1 shows the distribution of these diagnostic outcomes.

For DPE, 65.8% (812 samples) were obtained from fragments of lesions collected via biopsy procedures, 31.2% (384 samples) from biological material collected via scrapings of lesions, and 3.0% (38 samples) were not identified regarding material type. Regarding positive results, 52.8% (429/812) were from biopsy procedures and 43.3% (167/384) were from scraping.

Of the negative samples in the DPE that were forwarded for PCR confirmation, 24.4% (44/180) were from biopsy procedures and 30% (9/30) were from scrapings. Regarding the negative DPE samples that were not subjected to PCR, 45.7% (189) were from biopsy procedures and 47.8% (198) were from scrapings.

Among the patients diagnosed with TL, 423 (60.1%) were male and 281 (39.9%) were female. The ages of the patients ranged from 1 to 99 years, with a mean age of 42 years and a standard deviation of 19.9 years. TL cases are distributed according to the following age group: <10 (36 cases, 5.1%); 10–19 (83 cases, 11.8%); 20–39 (199 cases, 28.3%); 40–59 (242 cases, 34.4%); 60–69 (87 cases, 12.4%); 70–79 (46 cases, 6.5%); >80 (11 cases, 1.6%) Regarding pediatric patients, 36 (5.1%) were under 10 years old. Most cases were observed in individuals aged 20 to 59 years (441 cases, 62.7%), with the highest prevalence found in patients aged 50 to 59 years. Males were predominant across nearly all age groups.

Table 1 presents the number of positive tests in each registered region, expressed as a proportion of the total number of tests conducted. It also details the number of positive PCR results and of negative DPE results.

**Table 1:** Association between diagnostic methods for tegumentary leishmaniasis, positivity rates, and health regions from GAL/MG in Minas Gerais State, Brazil.

Health Region	DPE		PCR		DPE negative and PCR	
	N	Positive (%)	N	Positive (%)	N	Positive (%)
1	19	4 (21.1)	16	9 (56.3)	7	3 (42.9)
2	727	382 (52.5)	280	68 (24.3)	167	44 (26.3)
3	14	3 (21.4)	6	1 (16.7)	4	0
4	414	203 (49.0)	31	8 (25.8)	20	4 (20.0)
5	34	15 (44.1)	15	7 (46.7)	6	2 (33.3)
6	8	1 (12.5)	8	0	4	0
7	13	1 (7.7)	3	1 (33.3)	0	0
8	5	1 (20.0)	4	0	2	0

Data on the results of the diagnostic routine from 2017 to 2020 from the Laboratory Environment Management System of Ezequiel Dias Foundation for the diagnosis of cutaneous leishmaniasis; DPE = direct parasitological examination; PCR = polymerase chain reaction; N = number of examinations; 1 = Macro-region of Belo Horizonte and state health network of Belo Horizonte; 2 = North, Northeast, Northwest and Jequitinhonha; 3 = Southeast and Center-South; 4 = East, East of South and Steel Valley; 5 = West; 6 = South; 7 = North Triangle and South Triangle; 8 = Belo Horizonte municipality and the metropolitan region (GAL/MG Health regions).

**Figure 2:**
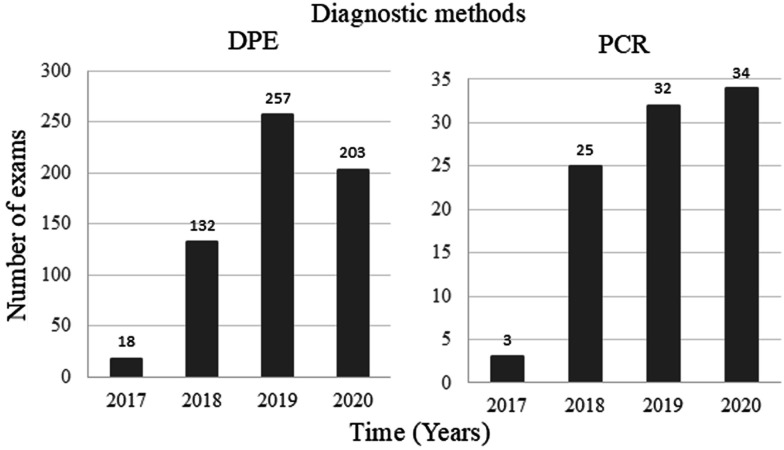
Temporal evolution of the number of cases of cutaneous leishmaniasis diagnosed by direct parasitological examination and polymerase chain reaction in Minas Gerais State, Brazil. Data on the results of the diagnostic routine from 2017 to 2020 from the Laboratory Environment Management System of Ezequiel Dias Foundation for the diagnosis of cutaneous leishmaniasis; DPE = direct parasitological examination; PCR = polymerase chain reaction.

The number of tests conducted and recorded in GAL/MG increased from 21 tests (comprising DPE and PCR) in 2017 to 499 in 2020, reflecting a 23.8-fold increase. Figure 2 depicts the temporal trends in the number of TL cases detected by DPE and PCR within the NPHL of MG.

## DISCUSSION

The development of a diagnostic network in MG is designed to facilitate early detection and timely treatment of TL cases, thereby mitigating morbidity. Minas Gerais State (MG) is the second most populous state in Brazil, with a population exceeding 20 million residents^8^. The region is endemic for TL and presents a well-established healthcare infrastructure. Consequently, the NPHL for TL diagnosis in MG has the potential to serve as a national model for effective disease management.

In this scenario, it is important to analyze the GAL/MG information system to evaluate the efficiency of a diagnostic network for TL. This is crucial because confirmed cases of the disease rely mainly on the results of DPE and/or PCR tests^11^.

The recommendation for employing DPE as the initial diagnostic tool is based on the rapid processing time, the possibility of performance at the point of care, and its high specificity^4^. Sample collection can be performed by any trained healthcare professional, and the assay does not require costly equipment or sophisticated laboratory infrastructure^5^.

Approximately 90% of suspected TL cases underwent DPE as the initial diagnostic test, yielding a positivity rate of 49.4%, likely attributable to the sensitivity of the method (ranging from 15% to 74.4%)^4^. It is not possible to confirm that all suspected cases were indeed TL, given the challenges in differentiating it from other clinically similar diseases^4^.

The integration of PCR into the diagnostic workflow for TL is intended to enhance diagnostic sensitivity. When parasitological examinations yield negative results, a subsequent PCR test should be performed to minimize false-negative diagnoses^12^. PCR demonstrates high specificity (ranging from 84% to 100%) and is regarded as an excellent confirmatory assay^11^.

In this study, only 41 patients (30.4%) were confirmed cases of TL using PCR as the initial diagnostic test, potentially due to the inclusion of patients who were not truly TL cases. PCR is frequently employed as the first diagnostic test in patients primarily suspected of mucosal leishmaniasis (ML), in which the parasitic load is generally lower, resulting in reduced sensitivity of both parasitological and molecular tests^13^. However, 53 patients (25.3%) were confirmed by PCR following a negative DPE, which highlights the importance of a second diagnostic test in these cases. The incorporation of PCR increases the sensitivity of cutaneous leishmaniasis diagnosis by 20% to 30% and of mucosal leishmaniasis diagnosis by 55% to 70% when compared with parasitological methods^13^. Therefore, PCR should be employed in patients suspected of TL when DPE presents negative results, or when species identification of *Leishmania* is required, thereby supporting accurate prognosis and enhancing disease surveillance efforts^14^.

Although biopsy is an invasive procedure for patients and an exclusive medical technique^5^, most biological samples collected were obtained via lesion fragment collected by biopsy procedures (65.8%).

The highest incidence rate was observed in males compared to females, with a predominance in the age group of 50 to 59 years. Despite the low number of cases reported in GAL/MG, these data approach those recorded in the Brazilian Notifiable Diseases Information System (SINAN) for the 2017–2020 period, in which 48,737 (73.9%) males and 17,230 (26.1%) females were recorded, with 43,504 (65.9%) cases reported in individuals aged 20 to 59 years and 4,288 (6.5%) cases reported in individuals under 10 years^2^.

Analysis of cases diagnosed within GAL/MG indicates that Health Region 2 (comprising North, Northeast, Northwest, and Jequitinhonha) recorded the highest number of DPEs, with 382 (62.6%) cases of TL from 2017 to 2020, followed by Region 4 (comprising East, Southeast, and Vale do Aco) with 203 cases (33.3%). This epidemiological profile is consistent with data from SINAN. In 2017–2020, Regions 2 and 4 recorded 2,990 (42,7%) and 1,420 (20,26%) cases of TL, respectively^2^. Regions with a higher number of suspected cases tend to report more confirmed cases and employ GAL/MG more frequently, reflecting their overall disease burden.

Despite the number of TL cases and records in GAL/MG increasing 23.8 times from 2017 to 2020 following the restructuring of the network, from our sample, a total of 414 patients (66.3%) with negative DPE results were not sent for PCR testing. This suggests a need to promote training to health professionals when facing the suspicion and flow of diagnostic procedures. The reduction in TL cases in 2020 may be attributed to the COVID-19 pandemic, which hindered patients’ access to the healthcare system^15^.

## CONCLUSION

Despite the inherent challenges in diagnosing TL, the NPHL in Minas Gerais State was found effective due to the employment of the initial test at the patient care site, which ensured easy access and timely treatment. Moreover, PCR emerged as an effective confirmatory method for cases with negative DPE results, enhancing diagnostic sensitivity by 25.3%. Therefore, the NPHL was demonstrated to be a promising model, suitable for expanding into similar diagnostic networks in Brazil, strengthening the TL surveillance and control program.

## References

[1] World Health Organization Leishmaniasis.

[2] Brasil. Ministério da Saúde Casos de leishmaniose tegumentar: Brasil, Grandes Regiões e Unidades Federadas, 2000 a 2022.

[3] Passos VM, Fernandes O, Lacerda PA, Volpini AC, Gontijo CM, Degrave W (1999). Leishmania (Viannia) braziliensis is the predominant species infecting patients with American cutaneous leishmaniasis in the State of Minas Gerais, Southeast Brazil. Acta Trop.

[4] Espir TT, Guerreiro TS, Naiff MF, Figueira LP, Soares FV, Silva SS (2016). Evaluation of different diagnostic methods of American Cutaneous Leishmaniasis in the Brazilian Amazon. Exp Parasitol.

[5] Goto H, Lindoso JA (2010). Current diagnosis and treatment of cutaneous and mucocutaneous leishmaniasis. Expert Rev Anti Infec Ther.

[6] Braz LM (2019). Tegumentary leishmaniasis diagnosis: what happened with MST (Montenegro Skin Test) in Brazil?. Rev Inst Med Trop Sao Paulo.

[7] Minas Gerais Secretaria de Estado de Saúde. Subsecretaria de Vigilância e Proteção à Saúde. Superintendência de Vigilância Epidemiológica, Ambiental e Saúde do Trabalhador. Diretoria de Vigilância Ambiental. Nota técnica CVRNB/DVA/SVEAST/sub.VPS-no011/2017. Estutura a rede estadual de laboratórios na rede SUS para diagnóstico parasitológico direto da leishmaniose tegumentar no estado de Minas Gerais.

[8] Instituto Brasileiro de Geografia e Estatística Cidades e estados.

[9] Barcia JJ (2007). The Giemsa stain: its history and applications. Int J Surg Pathol.

[10] Musso M, Wang JC, van Dyke MW (1996). In vivo persistence of DNA triple helices containing psoralen-conjugated oligodeoxyribonucleotides. Nucleic Acids Res.

[11] Pena HP, Belo VS, Xavier JC, Teixeira RG, Melo SN, Pereira DA (2020). Accuracy of diagnostic tests for American tegumentary leishmaniasis: a systematic literature review with meta‐analyses. Trop Med Int Health.

[12] Reithinger R, Dujardin JC, Louzir H, Pirmez C, Alexander B, Brooker S (2007). Cutaneous leishmaniasis. Lancet Infect Dis.

[13] Gomes AH, Martines RB, Kanamura CT, Barbo ML, Iglezias SD, Lindoso JA (2017). American cutaneous leishmaniasis: in situ immune response of patients with recent and late lesions. Parasite Immunol.

[14] Arevalo J, Ramirez L, Adaui V, Zimic M, Tulliano G, Sar C (2007). Influence of Leishmania (Viannia) species on the response to antimonial treatment in patients with American tegumentary leishmaniasis. J Infec Dis.

[15] Pan American Health Organization Leishmanioses: informe epidemiológico das Americas.

